# Exploring current conduction dynamics in multiferroic BiFeO_3_ thin films prepared via modified chemical solution method

**DOI:** 10.1038/s41598-024-76458-y

**Published:** 2024-10-26

**Authors:** Waseem Ahmad Wani, Harihara Venkataraman, Kannan Ramaswamy

**Affiliations:** 1 Department of Physics, BITS-Pilani, Hyderabad Campus, Medchal District, Hyderabad, Telangana 500078 India; 2 Department of Physics and Materials Centre for Sustainable Energy and Environment, BITS-Pilani, Hyderabad Campus, Medchal District, Hyderabad, Telangana 500078 India

**Keywords:** Bismuth ferrite, Chemical solution deposition, Leakage current, Space charge limited current, Energy science and technology, Materials science, Physics

## Abstract

The current study describes current conduction mechanisms in BiFeO_3_ thin films prepared by using a modified chemical solution-based technique. In particular, in these films, X-ray photoelectron spectroscopy revealed that the defect causing Fe^2+^ ions reduced by about 18% compared to the solution-based synthesis methods reported before, indicating better film quality. The leakage current density was measured to be 1.7 × 10^–6^ A/cm^2^ at an applied voltage of 1.5 V, which is one order of magnitude less than the previously reported work in a similar system. Oxygen annealing was found to be effective in further reducing the current conduction, making these films a suitable choice for device applications. The current–voltage curves exhibited three different behaviours of current conduction mechanisms in these films. In particular, when the applied electric field was increased, a transition from Ohmic conduction to trap-filled space charge limited conduction was observed. The presented investigations demonstrate the importance of deposition methods in determining the suitability of thin films for device applications.

**Supplementary Information:**

The online version contains supplementary material available at 10.1038/s41598-024-76458-y.

## Introduction

Over several decades, experts have worked to develop new avenues for energy conversion and storage to meet the ever-increasing demand for inexhaustible energy and cleaner fuel sources. Multifunctional oxide materials appear to be promising candidates for such applications due to their intriguing features. Among various oxide materials, oxide multiferroics have attracted keen interest due to the co-existence of several ferroic orders such as ferroelectricity, ferromagnetism and ferroelasticity^1,2^. Such materials provide additional degrees of freedom, which may either simplify the operation of existing device structures or provide new architectural options. Moreover, the presence of impurities and defect states (intrinsic or extrinsic) in these multiferroic materials profoundly influences their electrical and optical properties^3–5^. This makes it possible to use these materials for diverse applications such as solar cells, memory devices, sensors, and many more. Multiferroics have both spin ordering and electrical dipoles in the same material. As a result, these materials have got much attention for device applications and understanding of their fascinating physics.

Among several multiferroics, BiFeO_3_ (BFO) is probably the only multiferroic that exhibits a ferroelectric state at room temperature with high polarization up to 100 µC/cm^2^ and an antiferromagnetic ordering resulting in fascinating magneto-electric coupling^6^. BFO has attracted much attention because of its room temperature multiferroicity and ability to tune the properties using external parameters like ambient conditions (annealing temperature, annealing time, and ambient atmosphere), interfacial engineering, and defect engineering^7^. BFO is a member of the *R3c* space group and has a distorted rhombohedral perovskite structure (ABO_3_), with Bi atoms at the A-site and Fe atoms at the B-site. It exhibits high Curie temperature (T_.C._ ~ 1103 K) and high Neel temperature (T_N_ ~ 643 K). Besides these, BFO has a relatively higher optical absorption, piezoelectricity, and switchable photocurrent characteristics, all of which add to its versatility, making it an excellent candidate for multipurpose device applications^8,9^. Most notable among the accessible multiferroic materials, BFO stands alone owing to its relatively lower optical bandgap (2.1–2.7 eV), which fueled the interest of BFO towards photovoltaic applications^10–12^.

Despite these interesting features, the complexity in pure phase formation, along with the larger leakage current density, hinders practical device application of BFO. The higher leakage current density is primarily due to the oxygen vacancy defects and multiple valence states of Fe ions. The fabrication of a pure phase BFO device is complex since it is frequently associated with secondary phases (such as Bi_2_Fe_4_O_9_, Bi_25_FeO_40_) that are not always desirable^11,13^. However, physical vapour deposition (PVD) techniques [such as pulsed laser deposition (PLD), radio-frequency (R.F.) sputtering, and molecular beam epitaxy (MBE)] have dominated the scene when it comes to demonstrating high-quality BFO thin films for device applications^6,14,15^. On the other hand, the chemical solution deposition (CSD) technology is of great industrial relevance because of its cheap cost, precise control of the precursor composition, and simplicity of processing for large-area wafers^3^. However, achieving equivalent device performance and phase-pure BFO films using the CSD technique remains challenging. In response to these problems, we began optimizing the fabrication process of BFO thin films to achieve better photovoltaic performance. In this manuscript, we have focused on the electrical conduction of these films and the associated mechanisms rather than the photovoltaic properties.

Several research groups suggested using an excess of Bi (5–10 mol%) to eliminate the secondary phases mostly associated with the BFO films^16,17^. Our group previously reported the deposition of phase-pure BFO thin films using a solution deposition approach followed by spin coating processing without using the excess amount of Bi^3,18^ The deposition produced phase-pure BFO, but the amplitude of the diffraction peaks was substantially lower than that of films prepared using high-end techniques such as PLD, R.F. sputtering, and MBE. In this work, we present a different, cost-effective CSD technique which exhibited significantly higher diffraction amplitude, indicating the improved quality of the fabricated thin films. We believe that this preparation method will be helpful to researchers and industrialists who have been working to develop BFO-based devices. Moreover, we also present detailed investigations on the origin and mechanism of current conduction in the BFO films prepared using the new synthesis method reported here.

The manuscript is organized as follows. In “Fabrication and Characterization methods of BFO thin-films” section, the new method of fabricating BFO thin films and the experimental techniques used to characterize the obtained films are briefly described. This is followed by a section describing the structural, morphological, and optical bandgap estimation of the fabricated thin films. In “J–V curve measurements in BFO thin-films” section, results from the J–V curves in the BFO thin films are presented, and in “Current conduction mechanisms in the fabricated BFO thin-films” section, a detailed discussion about the current conduction mechanisms in these films in a range of applied electric fields is presented.

## Fabrication and characterization methods of BFO thin-films

In the chemical synthesis procedure to obtain BFO, bismuth nitrate pentahydrate and iron nitrate nonahydrate were used as starting materials. For the first time in this work, we used ethanolamine as a chemical agent to prepare BFO films. The addition of ethanolamine, in combination with other chemicals, has proven to be a distinctive factor in achieving a single phase of BFO films. This chemical agent has resulted in forming a pure phase of BFO and producing high-quality films, as evidenced by the XRD diffraction patterns and improved electrical properties.

Ethanolamine and 2-methoxy ethanol were used as stabilizers and solvents, respectively. To begin with, stoichiometric ratios of bismuth nitrate pentahydrate (4.8507 g, having a molecular weight of 485.07 g/mol, SRL chemicals) and iron nitrate nonahydrate (4.04 g, having a molecular weight of 404.00 g/mol, SRL chemicals) were dissolved in a mixed solvent of ethanolamine (Sigma Aldrich) and 2-methoxyethnol (20 mL, sigma Aldrich) using a magnetic stirrer. (i.e., we used 1.2216 g of ethanolamine with a molecular weight of 61.8 g/mol). Nitric acid (10 mL, TCI) was added to the solution while stirring it at room temperature for an hour. The precursor solution was then transferred to a heated plate and aggressively stirred for 40 min at 120°C until it became dark red. The resulting solution was aged for about 12 h before the actual deposition process. The spinning rate, duration, acceleration time, and quantity of coatings were all tuned to deposit a pure, uniform, crack-free, and compact film. Before the actual annealing procedure, the prepared films were pre-fired for 15 min (5 min each at three distinct phases) at 300°C. Finally, the produced films were annealed in an air and oxygen atmosphere for 30 min in a tube furnace at 600°C (supplementary Fig. 1). The key difference between the current samples and those in previous studies is the use of ethanolamine as a solvent precursor, which significantly improved phase purity, crystallinity, and overall film quality^3^.

**Fig. 1:**
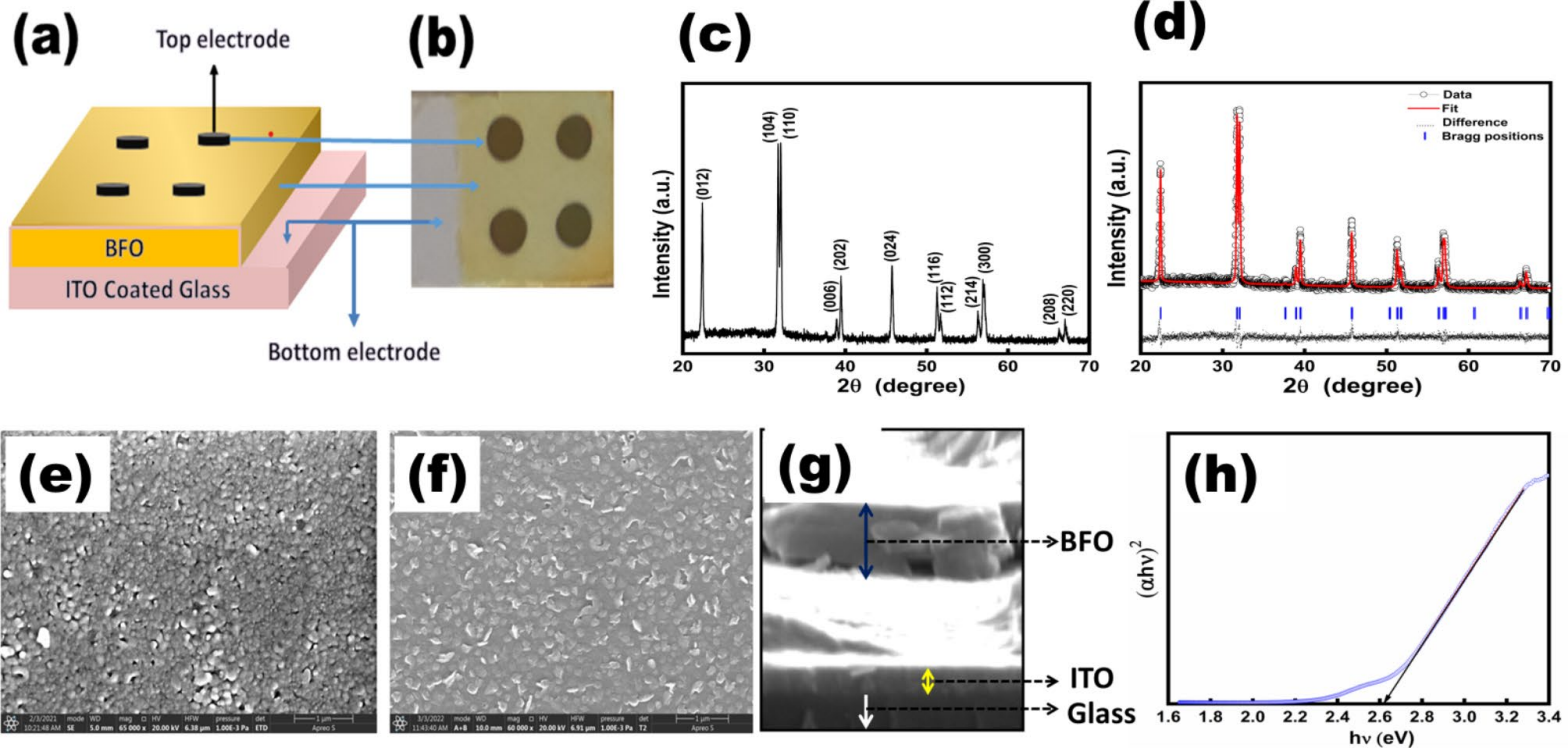
(**a**) Schematic architecture of fabricated thin-film devices, (**b**) Actual picture of the prepared device, (**c**) XRD pattern of BFO film, (**d**) Rietveld refined XRD data, (**e**) SEM image of old BFO film, (**f**) SEM image of BFO film under investigation, (**g**) cross-sectional SEM image of BFO film, (**h**) bandgap determination of BFO film.

The e-beam deposition technique was employed in conjunction with circular shadow masks to deposit the top Al-electrodes of the area (7.07 mm^2^) for J –V curve measurements. An X-ray diffractometer (Rigaku Ultima-IV, Cu-kα radiation source, λ = 1.5406 Å) was used to determine the crystalline structure. The surface morphology, film thickness, and film compactness were examined using field emission scanning electron microscopy (FESEM, Apreo LoVac). X-ray photoelectron spectroscopy was used to assess the valence states of the components (Thermo Scientific Cu k Alpha). The XPS spectra were calibrated using the standard C 1 s peak at 284.8 eV to ensure accurate binding energy measurements. A UV–visible spectrophotometer was used to obtain the diffuse reflectance spectra of the prepared films to determine the electronic bandgap (Jasco, UV-670, FP-6300). Finally, the current conduction was measured using a Keysight (B2912A) source meter. Figures 1a and b illustrate our device’s schematic configuration and real picture, respectively.

## Structural, morphological, and optical bandgap of BFO thin-films

The high-quality polycrystalline nature of the BFO films was confirmed by X-ray diffraction (XRD) patterns, as given in Fig. 1c. Rietveld refinement of the prepared film revealed that these films exhibit rhombohedrally distorted perovskite phase and are free of impurity phases, as shown in Fig. 1d. The extracted lattice parameters indicated are given in Table 1. Moreover, it appeared from these patterns that all the diffraction peaks have higher intensity in comparison to the reported literature^3,18^. An increment of 16-fold in the intensity value of (110) peak was observed by adopting the current deposition technique, which is significantly more than our previously reported investigations of BFO thin films prepared by CSD technique 19, indicating improved quality of prepared films. Additionally, the FESEM pictures (taken at different locations) demonstrated a compact and smooth surface with a uniform spread of the granular shapes and sizes, indicating that the films are well-grown on ITO/glass substrates. Figures 1e–g depict the prepared films’ surface morphology and cross-sectional images with a thickness of about 370 nm. The bright gap between BFO and ITO, as seen in Fig. 1g, is probably due to the non-uniform cut of the sample. The cross-sectional image was obtained by breaking the sample using a glass cutter, followed by manual hand pressing. This method, while employed for sectioning, has inherent limitations, and it seems that it resulted in a non-uniform cross-sectional cut of the sample. The contrast observed in the image is likely attributable to variations in the heights of the ITO and BFO layers in relation to the electron source. Moreover, the UV–vis-NIR absorbance spectrum revealed a direct optical bandgap of 2.61 ± 0.03 eV, as shown in Fig. 1h, which agrees with the reported values^18,19^.

**Table 1:** Structural properties obtained from Rietveld refined analysis.

BFO—Space group R3C (161), lattice parameters a = 5.582 Å c = 13.877 Å												
hkl planes	012	104	110	006	202	024	116	112	214	300	208	220
Peak position (2θ)	22.413	31.717	32.042	38.910	39.440	45.720	51.258	51.700	56.297	57.146	66.290	67.001
Interplanar spacing (Å)	3.9635	2.8188	2.7910	2.3127	2.2826	1.9828	1.7808	1.7666	1.6328	1.6105	1.4087	1.39558
Particle size (Å)	494	505	543	514	450	445	430	509	504	480	535	580

## J–V curve measurements in BFO thin-films

The larger current conduction in the BFO thin films dramatically diminishes their practical optoelectronic applications. As a result, several efforts have been undertaken to lower the leakage current of BFO thin films. Among them, cation substitutions at the Fe site of the BFO are effective in lowering leakage current to a considerable degree^3,20^. However, enhancing the film quality is another important aspect of controlling leakage current density. For example, our previous work found one order-of-magnitude drop in leakage current density by annealing the samples in an oxygen-rich environment^18^. Usually, high-end device fabrication techniques (such as PLD, MBE, and RF-sputtering) result in very efficient device performance^6,14,21^. However, we obtained a comparable device performance of these thin films by employing an inexpensive CSD technique.

**Fig. 2:**
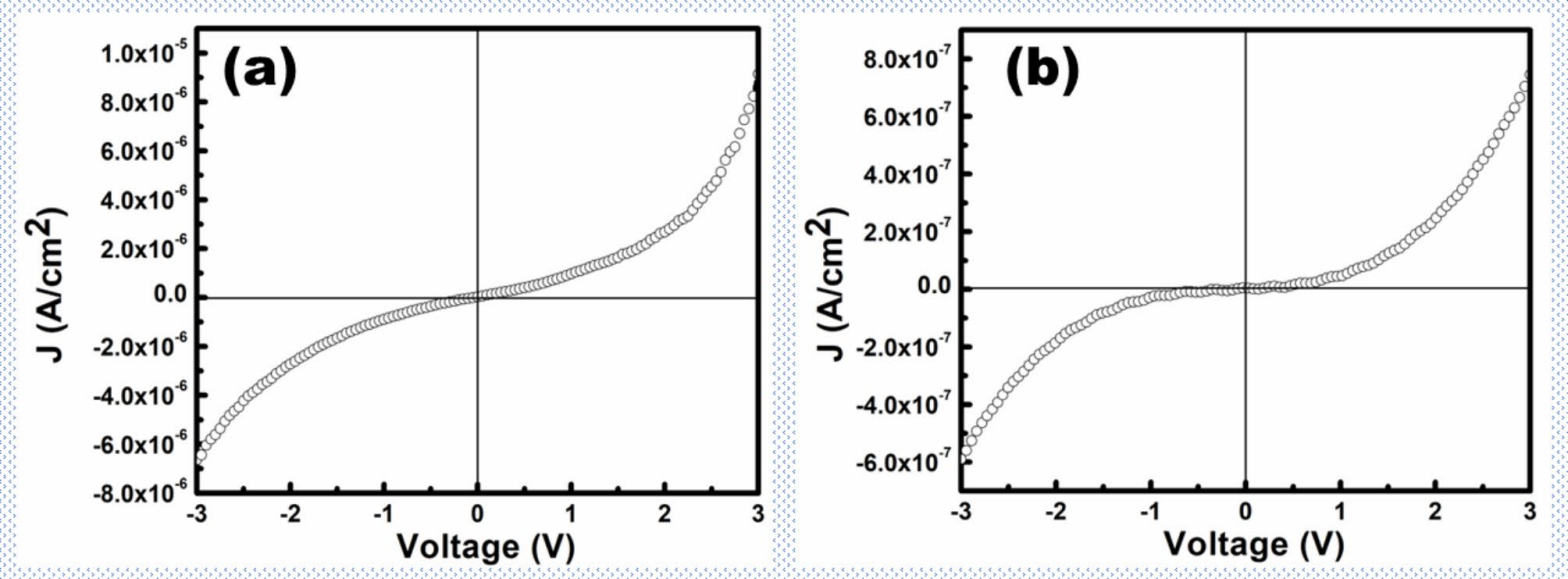
Current density of BFO films annealed in (**a**) air, (**b**) oxygen.

Figures 2a, b show the J-V curves of BFO samples annealed in air and oxygen atmospheres upon applying higher voltages (-3 V to + 3 V). The leakage current density of the BFO films under investigation was one order of magnitude lower than that stated in the literature. The current density of the BFO new and BFO old measured at room temperature at a voltage of 1.5 V was 2.2 × 10^–5^ A/cm^2^ and 1.7 × 10^–6^ A/cm^2^, respectively^3^.

Moreover, as can be seen from these J-V curves, the leakage current density is reduced by two orders of magnitude upon oxygen annealing, indicating that the existence of oxygen vacancies is the primary reason for the poor insulating property of BFO thin films. These results are in line with our previous work^18^. The overall J-V curve depicted multiple behaviours, owing to current conduction’s different origins, indicating a competitive conduction mechanism in which different conduction models dominate in different applied electric field regions. As can be seen from Fig. 2, the J-V curve appears to be linear up to a certain applied field in air-annealed films (Fig. 2a), whereas no such region of linearity appears in oxygen-annealed films. These results suggest that there exists different origins of current conductions in these devices.

## Current conduction mechanisms in the fabricated BFO thin-films

**Fig. 3:**
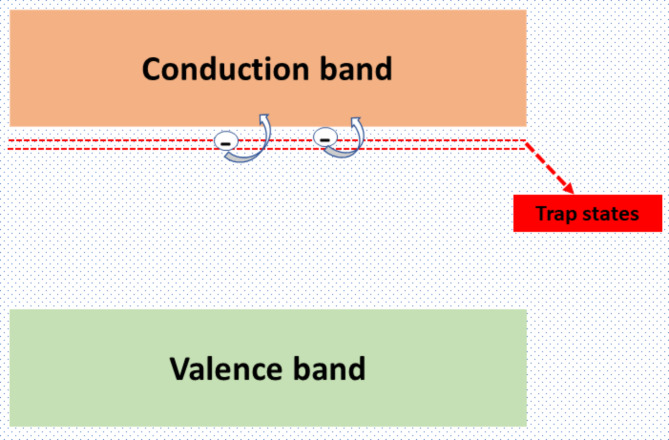
Pictorial representation of free carriers in BFO.

Current conduction in the BFO films is believed to be mostly driven by lattice defects such as oxygen vacancy defects and the presence of Fe^2+^ ions. The existence of oxygen vacancies is inevitable due to the annealing process in air. Likewise, the presence of lower-charged impurities of Fe^2+^ states is unavoidable due to their natural stability^20,22,23^. Moreover, the generation of these defects can be viewed as a mutual causality, in which one causes the other and vice versa, resulting in the existence of both Fe^2+^ and oxygen vacancies to guarantee charge neutrality. The co-existence of both kinds of defects is essential in describing the multiple current conduction mechanisms in these devices. The higher current conduction in these devices compared to other ferroelectric oxide-based devices (for example, the leakage current density in BaTiO_3_-based devices is significantly lower) is mainly ascribed to the higher charge carrier concentration generated due to oxygen vacancies^24^. Furthermore, we believe that the current conduction in these devices can be understood by invoking both intramolecular and intermolecular conductions originating from free charge carriers and virtual hopping of Fe^2+^ ions. The intra-molecular current conduction in BFO originates due to oxygen vacancies. Each oxygen vacancy leaves behind two free electrons. Therefore, the higher the concentration of oxygen vacancies, the more free carriers the film will generate, which in turn decides the leakage current density in these devices. These free electrons usually lie in shallow trap states below the conduction band, as depicted in Fig. 3. These trap levels in the forbidden band of BFO are generated by oxygen vacancies and are located 0.6 eV below the conduction band edge. Therefore, the trapped electrons can easily excite from these trap states to the conduction band and carry the current upon applying a small external field. i.e., electrons from the oxygen vacancies can be emitted much more easily into the conduction band than those from the valence band. Owing to the higher conductivity and linearity of the J-V curve in the air-annealed films, the current conduction is believed to originate from the higher concentration of charge carriers generated by oxygen vacancies^9,20,25,26^.

**Fig. 4:**
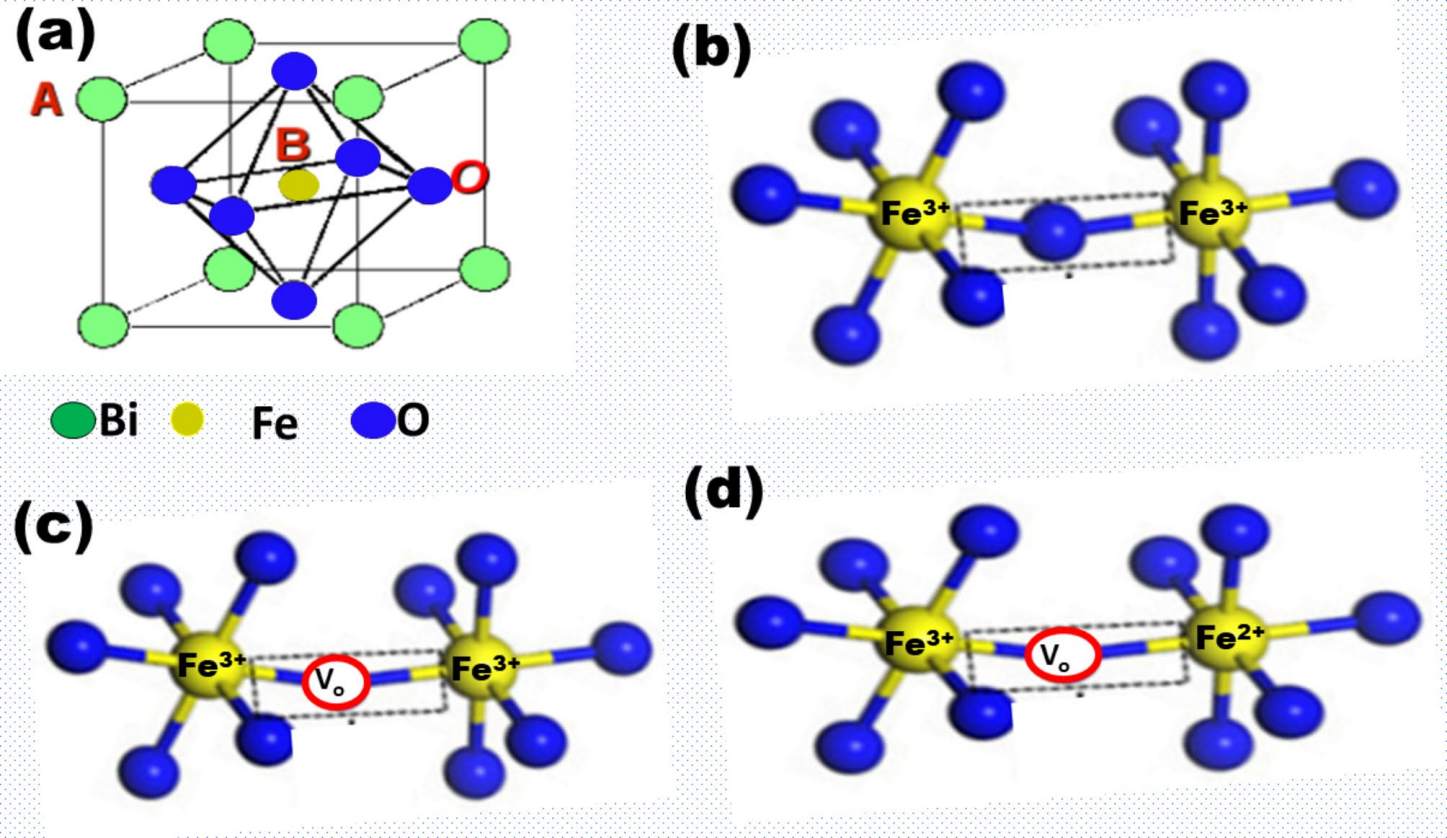
Pictorial representation of (**a**) ABO_3_ type BFO, (**b**) two FeO_6_ octahedrons are linked by oxygen atoms, (**c**) two FeO_6_ octahedrons are linked by oxygen vacancy, (**d**) two FeO_6_ octahedrons (one with Fe^3+^ and other with Fe^2+^) are linked by oxygen vacancy.

Now, the intermolecular current conduction in BFO can be conceived via a virtual ion hopping mechanism, i.e., the hopping of Fe^2+^ ions from one lattice site to the other. The co-existence of both kinds of defects (i.e. oxygen vacancies and Fe^2+^) is essential to understanding the hopping conduction mechanisms. The polaron hopping conduction mechanism has been well understood in ferric materials, such as Fe_3_O_4_, where Fe^2+^ occupies an octahedral site, and Fe^3+^ occupies both octahedral and tetrahedral sites. Since octahedrons and tetrahedrons share faces, electrons may easily move from Fe^2+^ to Fe^3+^^27^. In order to understand this mechanism in BFO-based devices, we need to understand the structure of BFO. As we know, BFO exhibits rhombohedrally distorted perovskite structure (ABO_3_ type), with Bi at the A-site and Fe at the B-site, as shown in Fig. 4a. In such structures, the two FeO_6_ (with Fe at the center and O atoms at the vertices) octahedrons are linked by oxygen atoms, as given in Fig. 4b. Therefore, the direct hopping of Fe^2+^ ions is not permissible. However, polaron hopping becomes conceivable in these materials if oxygen vacancies exist. Let us assume two octahedrons linked by an oxygen vacancy defect, as depicted in Fig. 4c. The presence of oxygen vacancy demands charge compensation, which can be achieved by converting Fe^3+^ to Fe^2+^. Let’s consider a situation where the oxygen vacancy bridges the Fe^3+^ and Fe^2+^ ionic states, as given in Fig. 4d. The oxygen vacancies bridge the electron transfer between Fe^2+^ and Fe^3+^ ions as these behave as ineffective electron trap centers. The oxygen vacancies act as positively charged canters and attract electrons from Fe^2+^, quickly transferring them to Fe^3+^ ions due to their (oxygen vacancy) ineffective electron-holding capability. Based on this, it is plausible to presume that Fe^2+^ hops (although virtually) from one lattice site to the other. Therefore, the co-existence of Fe^2+^ ions and oxygen vacancies (although lesser in concentration) is essential in realizing intermolecular current conduction in such devices. As clearly seen from Fig. 2b, since there is no linearity region in oxygen-annealed films, it is plausible to presume that polaron hopping (although virtually) from Fe^2+^ to Fe^3+^ plays a dominating role in deciding the current conduction of oxygenated BFO thin film devices^20,28^.

**Fig. 5:**
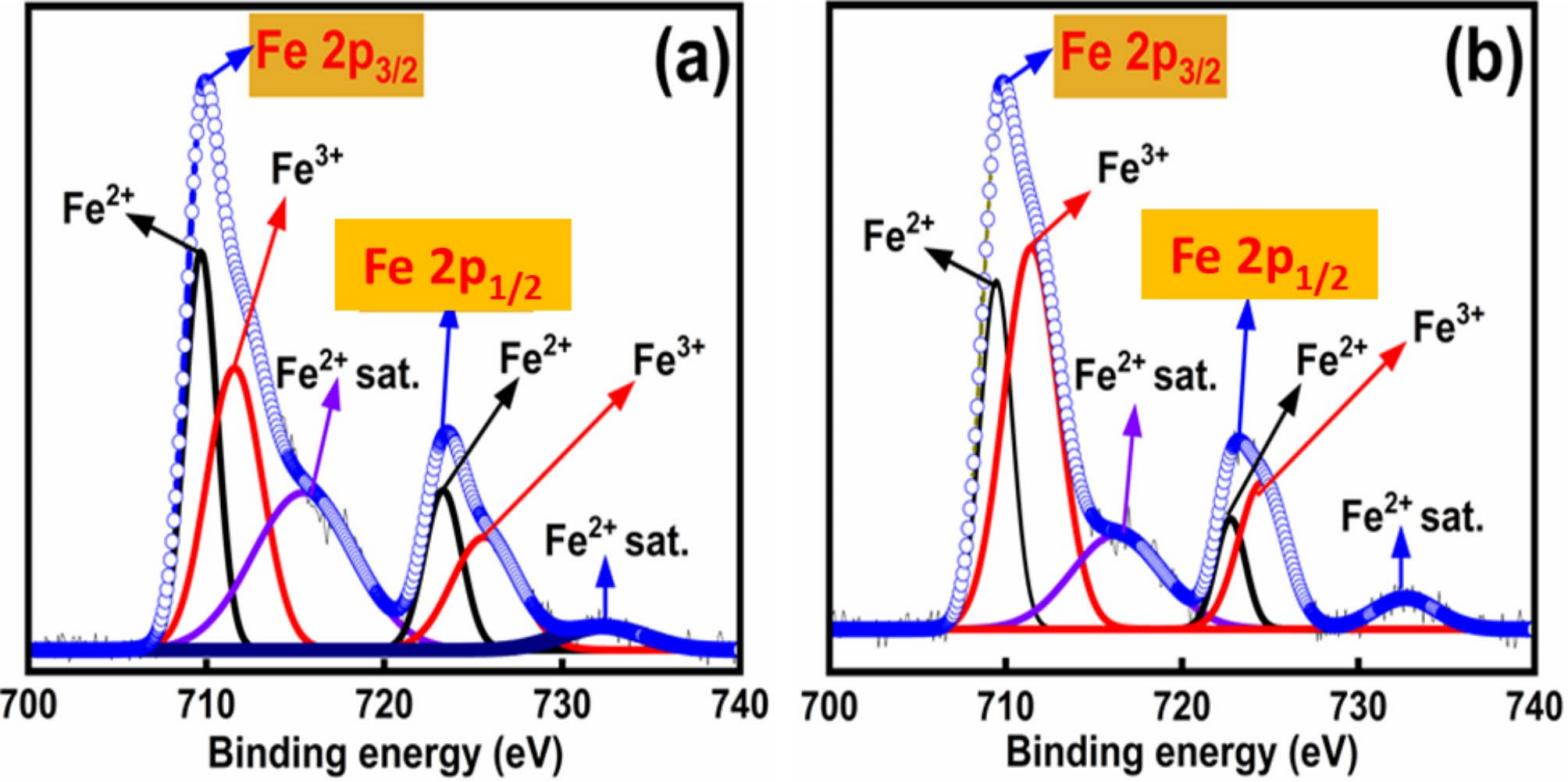
Fe-2p XPS spectra of (**a**) old BFO film, (**b**) BFO film under investigation.

In order to validate the above results, we used X-ray photoelectron spectroscopy to confirm the valence states of prepared thin films’ constituents. For comparative analysis regarding the concentration of Fe^2+^ states, we plotted the Fe 2p spectra of samples under investigation and the previously reported work. These spectra are presented in Fig. 5 (XPS spectra of O 1 s and Bi 4f. in supplementary Fig. 2). The positions of Fe 2p_3/2_ are anticipated to be at 710.7 eV for Fe^3+^ and 709.4 eV for Fe^2+^ states^23^. The Gaussian fitting of the Fe 2p spectra confirmed the co-existence of Fe^2+^ and Fe^3+^ states in the deposited films. On the other hand, the concentration of the Fe^2+^ states was significantly lower than in the previous study. The ratio of Fe^2+^: Fe^2+^ states in this study and the previous one was estimated to be 1:1.3 and 1:1.1, respectively. The concentration of Fe^2+^ states was found to reduce further (~ 1.4) upon oxygen annealing. Therefore, the reduction in Fe^2+^ amount (thus oxygen vacancies) could be the possible reason for the observed lower leakage current density^17,22,26–28^. These results lend support to the origin of the current conduction discussed above.

Coming towards the current conduction in BFO-based thin-film devices, the mechanism of charge transportation is still not clear. Because of this uncertainty, different charge transport models are used to back up the results of experiments on BFO devices. Table 2 describes the several models that have been reported in the literature. Among these charge transport models, the space charge limited current conduction (SCLC) model is one of these devices’ most well-known and widely accepted charge transport mechanisms.

**Table 2:** Various conduction mechanism models reported in the literature.

Sample	Low field	High field	Deposition method	References
BiFeO_3_	Poole–Frenkel	Poole–Frenkel	RF	^29^
BiFeO_3_	Poole–Frenkel	Poole–Frenkel	PLD	^30^
BiFeO_3_	Poole–Frenkel	Fowler-Nordheim tunneling	PLD	^31^
BiFeO_3_	SCLC	SCLC	CSD	^32^
BiFeO_3_	Ohmic conduction	SCLC	PLD	^20^
BiFe_0.95_Mn_0.05_O_3_	Ohmic conduction	Ohmic conduction	CSD	^32^
Bi_0.92_La_0.08_Fe_0.95_Mn_0.05_O_3_	Ohmic conduction	SCLC	RF	^15^
Bi_0.92_La_0.08_ FeO_3_	SCLC	SCLC	RF	^15^
BiFe_0.95_Ti_0.05_O_3_	Ionic Conduction	Ionic conduction	PLD	^20^
BiFe_0.95_Ni_0.05_O_3_	Ohmic conduction	SCLC	PLD	^20^

**Fig. 6:**
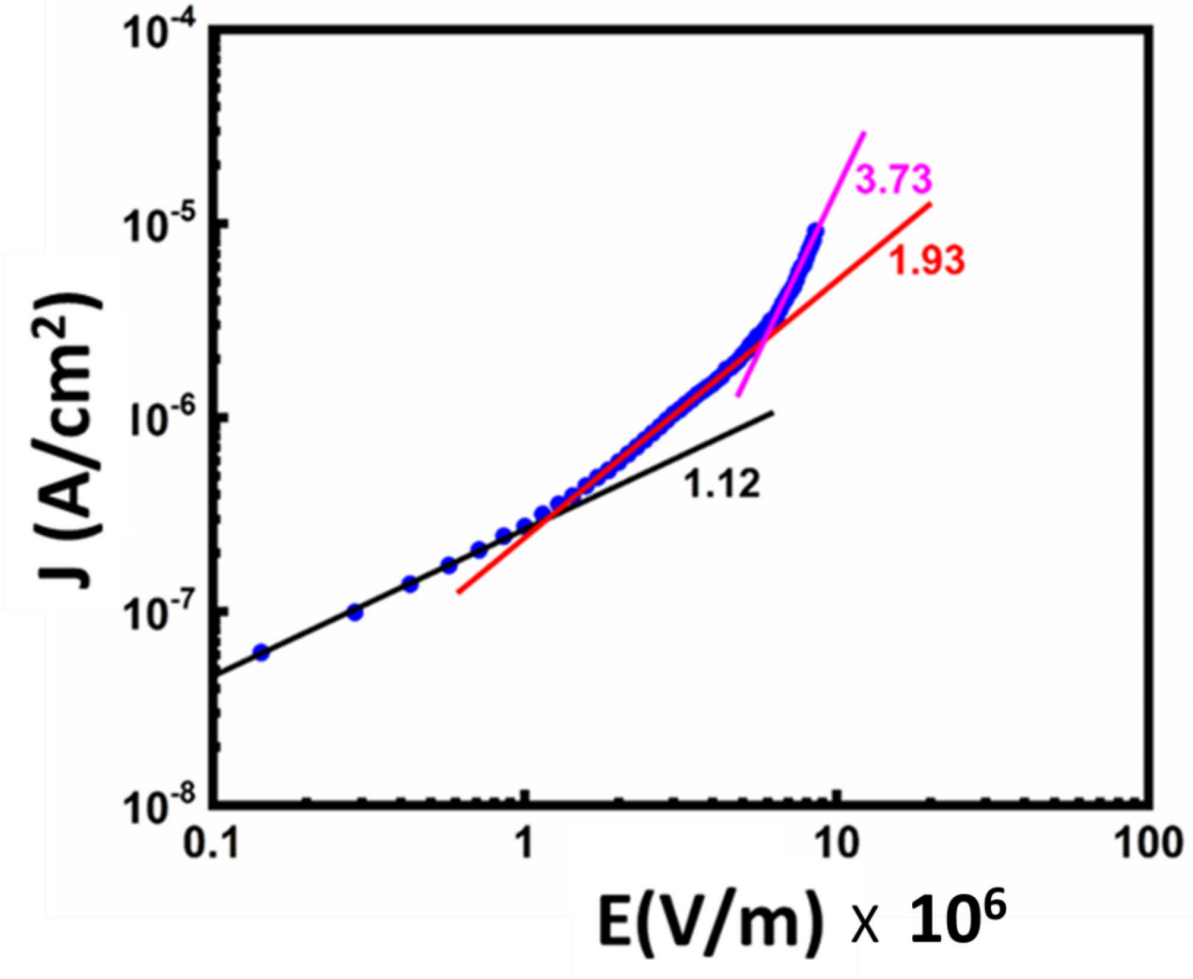
Logarithmic graph of J-E curve of BFO film.

To understand the leakage current mechanism in BFO, we considered the air-annealed films. Therefore, the term BFO implies BFO annealed in normal air conditions. We considered several models further to investigate the nature of current conduction in these films. However, we noticed that the obtained results fit nicely with the SCLC model only up to a certain applied field. According to this model, the current density behaviour may be subdivided into several zones. The slope of each zone (log J versus log E) specifies the conduction mechanism as given in Fig. 6. Therefore, we plotted the logarithmic behaviour of J with the applied electric field (E) and observed that the overall curve could be analyzed with three conduction behaviours in different applied field regions, as shown in Fig. 6. The corresponding slopes in these regions were found to be 1.12, 1.97 and 3.7, respectively as pictorially represented in Fig. 9c. The applied field values at E_tr_ and E_TFL_ were found to be 0.95 × 10^6^ V/m and 4.4 × 10^6^ V/m, respectively, where E_tr_ is the applied electric field at which current undergoes transition from O.C. to SCLC and E_TFL_ is the applied electric field at which current undergoes transition from SCLC to trap-filled limited SCLC mechanism.

**Fig. 7:**
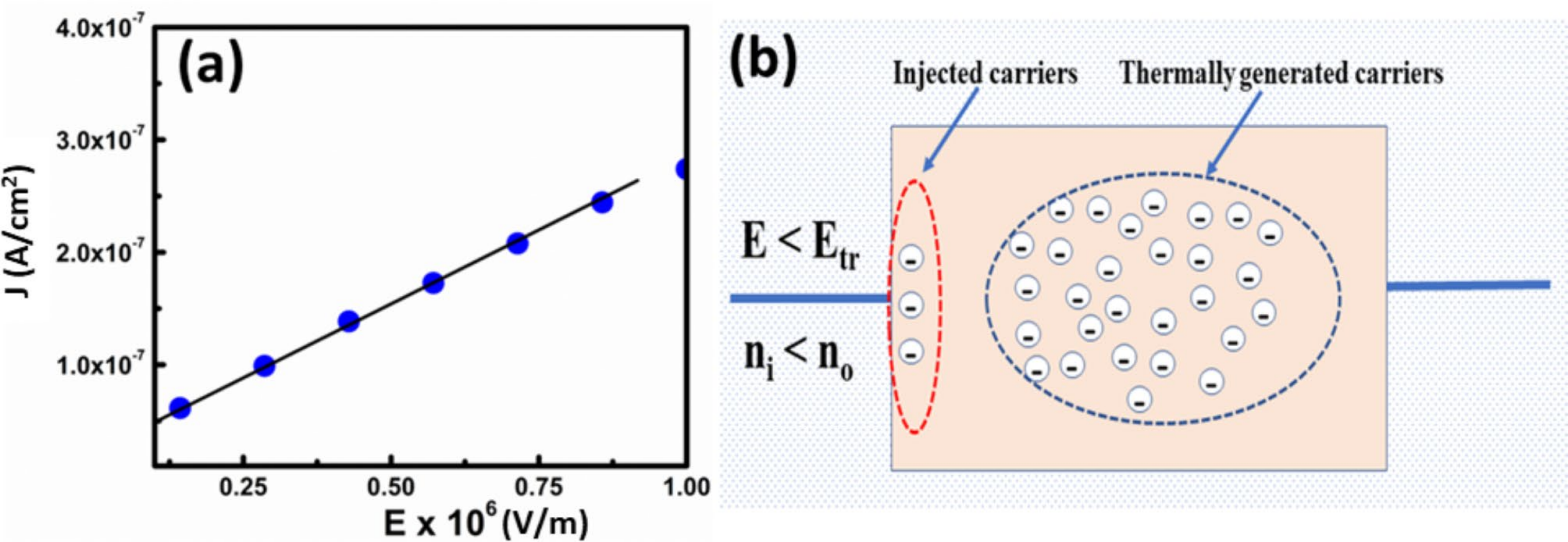
(**a**) Linear fitting of J-E curve at electric fields between (0.25 – 1.0)*10^6^ V/m (**b**) Schematic model of carrier distribution in BFO film at lower injected carrier density (E < E_tr_).

Since the SCLC effect dominates only when the injected carrier concentration (**n**_**i**_) exceeds the equilibrium carrier concentration (**n**_**o**_), thus, at lower fields (E < E_tr_), since n_o_ > n_i_, the curve exhibited almost linear behaviour up to E = 0.95 × 10^6^ V/m, with the slope value of 1.12, as shown in Fig. 7a. Since the m value is close to unity, these results suggest that Ohmic conduction (J α E^m^, m ~ 1) dominates at fields less than ~ 10^6^ V/m. In this region, the thermally generated electrons which occupy the trap centers in the material get excited to the conduction band and are responsible, thereby carrying current upon applying the external field of about 10^6^ V/m for the current. The schematic of the conduction mechanism is depicted in Fig. 7b. In particular, the schematic portrays that at lower injected carrier electron density (electrons inside a red-bordered vertical ellipse), the thermally excited electrons (electrons within the blue-bordered ellipse), which occupy the trap centers denoted as a minus sign inside a small circle are responsible for the current.

**Fig. 8:**
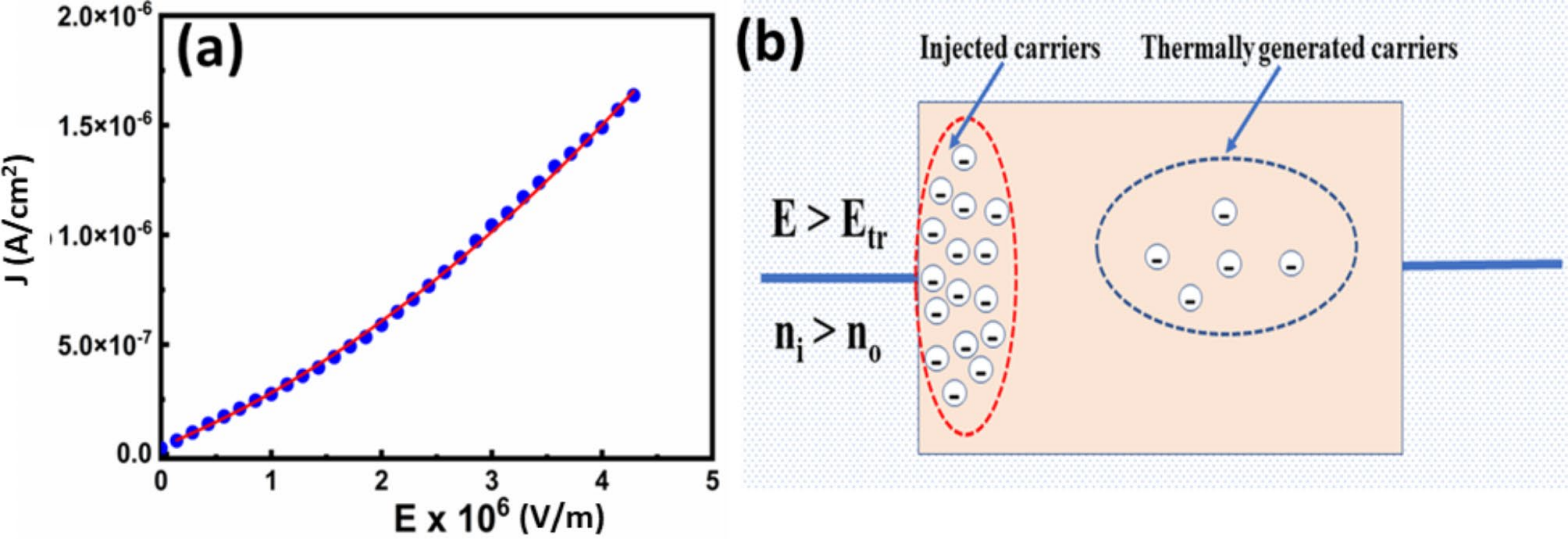
(**a**) Fitted J-V curve with modified Langmuir-Child law, (**b**) Schematic model of carrier distribution in BFO film under moderate carrier injection in space-charge-limited conduction (E_TFL_ > E > E_tr_).

However, at higher fields (E_TFL_ > E > E_tr_) (from 0.96 × 10^6^ V/m to 4.4 × 10^6^ V/m), the graphs exhibited a considerable divergence from linear behaviour (J α E^m^, m > 1), and the obtained value of m was found to be 1.97, indicating that the SCLC mechanism is dominant in this region. Actually, the curve in this region follows the modified Langmuir-Child law (J = aE + bE^2^) as given in Fig. 8a, thereby confirming the SCLC mechanism is predominating. In this region, the concentration of injected electrons has substantially surpassed the equilibrium concentration (n_i_ > n_o_) in the thin film, contributing to the rise in leakage current as schematically presented in Fig. 8b.

**Fig. 9:**
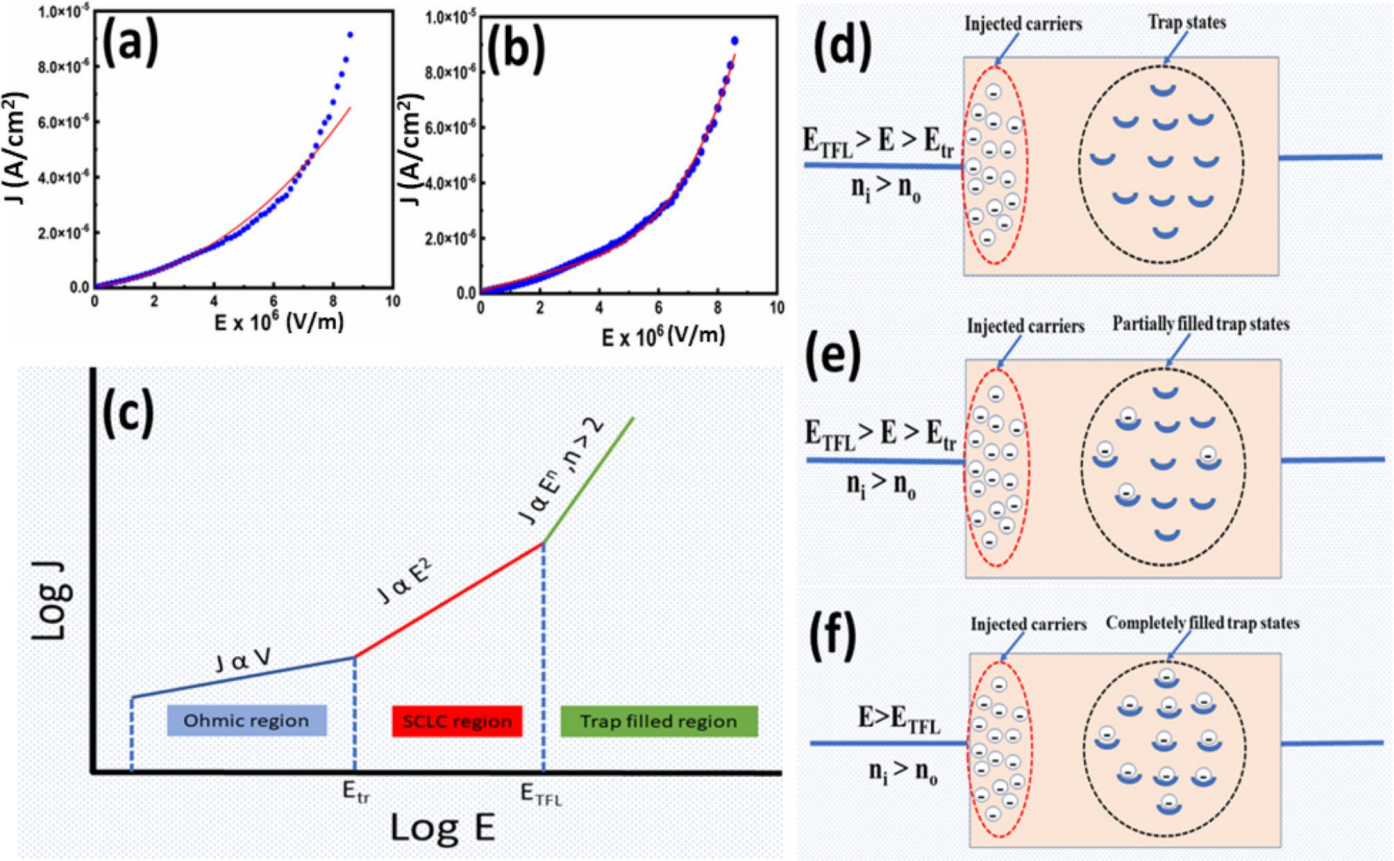
(**a**) Langmuir–Child fitting of the J–E curve at the higher field (polynomial fitting of order 2), (**b**) polynomial fitting of order 4, (**c**) pictorial representation of Lampert conduction, and schematic model of carrier distribution in BFO thin-film film under moderate carrier injection in space-charge-limited conduction at higher injection (E > E_TFL_) with (**d**) unfilled traps, (**e**) partially filled traps, (**f**) completely filled traps. Note that the traps are filled by the thermally generated electrons excited from the valence band in the absence of any applied bias.

Furthermore, we also attempted polynomial fitting of order two in the entire applied field region, as shown in Fig. 9a, but the fitted curve showed much variation. However, when we fitted the data using the polynomial fit of order 5, the curve fitted nicely, as depicted in Fig. 9b. These results indicate that the standard SCLC mechanism cannot fully explain the conduction behaviour at higher fields; therefore, further investigations are required to understand the conduction behaviour at higher fields.

As can be seen from Fig. 6, at higher applied fields (E > E_TFL_) (beyond E = 4.4 × 10^6^ V/m), an abrupt rise in current occurs. The m value reached 3.7, which is much higher than to satisfy the Langmuir-Child law, indicating the possibility of a trap-filled limited conduction mechanism in these samples. Since the **m** value of the curve in this region was found to be 3.7, as indicated in Fig. 6, such behaviour can be explained by Lampert’s triangle, as demonstrated in Fig. 9c^33^. (i) At lower fields, the carrier concentration of thermally generated charge carriers is greater than that of injected charge carriers, resulting in Ohmic conduction. (ii) The injected carrier concentration surpasses the thermally stimulated carriers at higher fields. When the number of injected carriers exceeds the number of background carriers (thermally generated carriers), the injected carriers spread and form a space-charge field. This field controls the currents, known as space charge limited currents (SCLCs) and obeys the square law as shown in Fig. 9c. However, if the sample contains traps (as shown in Fig. 9d, some of the injected carriers will be trapped while others will stay free, as depicted in Fig. 9e. As the applied field grows, so does the number of injected charge carriers, and more and more traps are filled with carriers. At E_TFL_, since all the traps are completely filled, there is an abrupt rise in current conduction, as demonstrated in Fig. 9f^34^. As discussed above, these trap states are mostly the defect levels generated by oxygen vacancies.

Moreover, as evident from Fig. 2, the forward and reverse bias currents vary, which indicates that the electrode-limited current conduction mechanism also contributes to the conduction process. The variation in forward and reverse bias currents could be due to different work functions, carrier concentrations and electron mobilities of the respective electrodes^18^. An electrode-dependent J-V data is suggested to verify the role of the electrodes on the current conduction processes. However, the results suggest that a trap-filled limited current conduction mechanism is the dominating conduction process in BFO-based thin film devices prepared by the modified chemical solution deposition technique.

## Conclusions

In conclusion, highly crystalline BFO thin films were deposited using low-cost and simple CSD techniques. The current conduction reveals a transition from Ohmic conduction to a trap-limited SCLC mechanism with an increasing electric field. The XPS findings imply the co-existence of Fe^2+^ and Fe^3+^, indicating the virtual hopping conduction in these devices. The higher leakage current conduction is believed to originate from the inter-molecular and intra-molecular contributions, interlinked with the existence of oxygen vacancies. These undoped films’ relatively lower leakage current density makes them attractive for practical device applications.

## Electronic supplementary material

Below is the link to the electronic supplementary material.

Supplementary Material 1

## Data Availability

The data that support the findings of this study are available from the corresponding author upon reasonable request.
