# Supplementary material for: Exploring current conduction dynamics in multiferroic BiFeO3 thin films prepared via modified chemical solution method
Source: Sci Rep. 2024 Oct 26;14:25578. doi: 10.1038/s41598-024-76458-y (PMC11513044; doi:10.1038/s41598-024-76458-y)
Supplement: Supplementary file 1 — Supplementary Material 1 [file 41598_2024_76458_MOESM1_ESM.docx]

**Exploring Current Conduction Dynamics in Multiferroic BiFeO₃ Thin Films Prepared via Modified Chemical Solution Method**


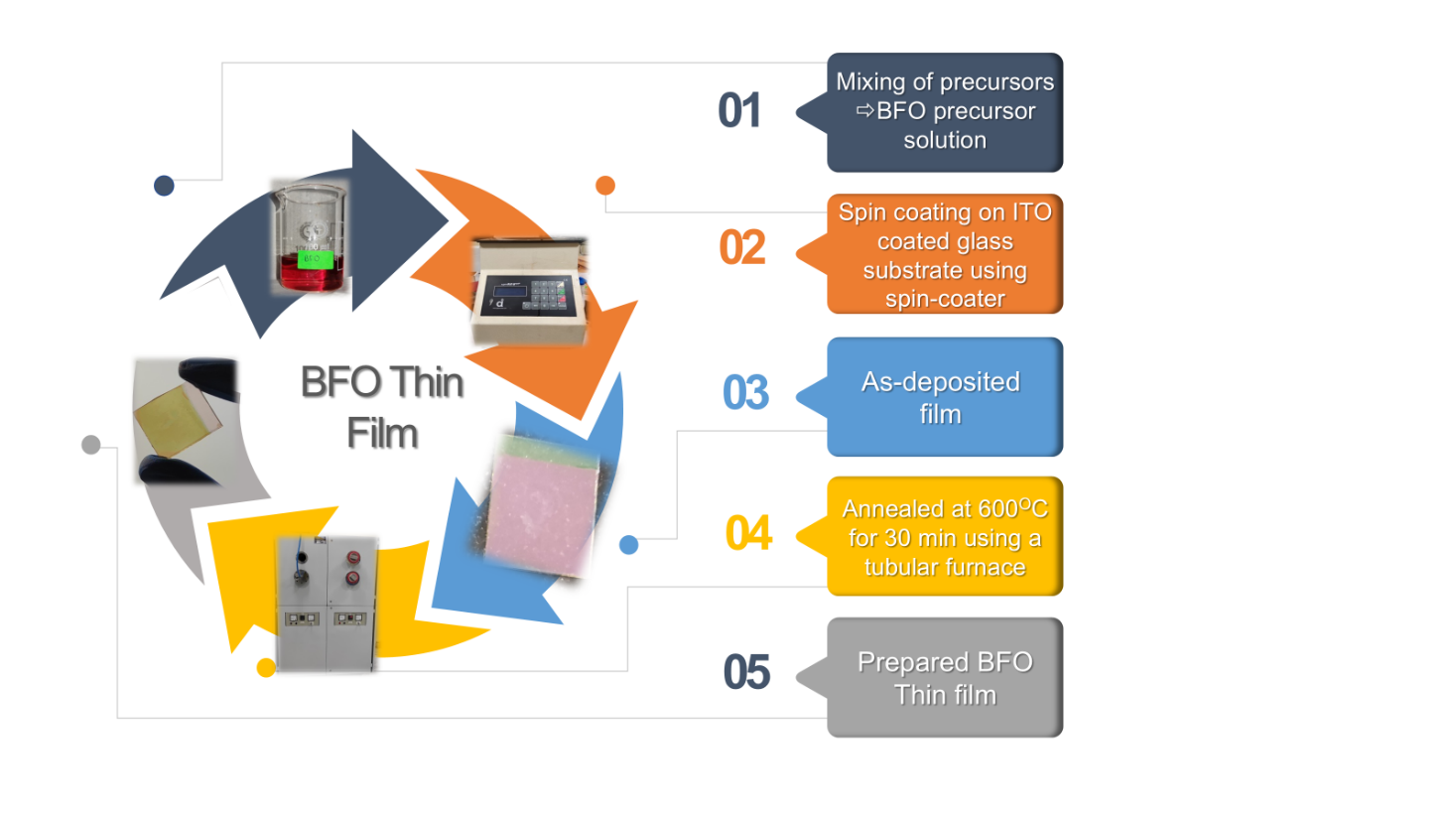


Figure 1


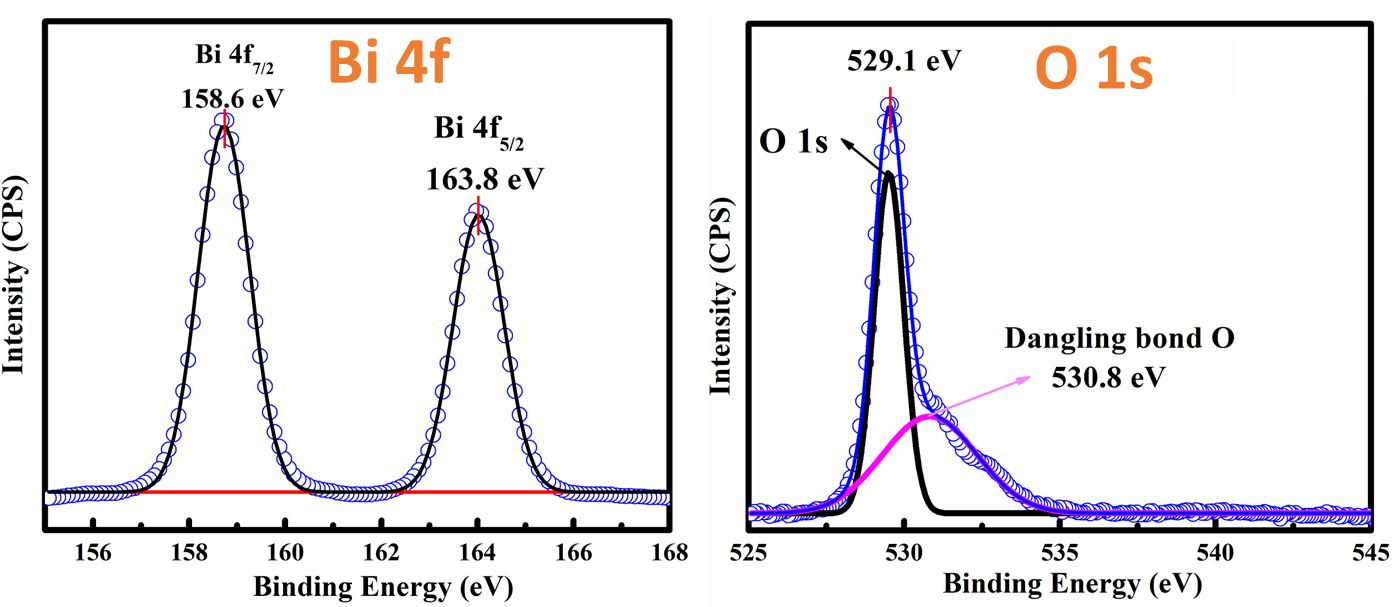


Figure 2
